# The influence of the COVID-19 pandemic, sex, and age on temporomandibular disorders subtypes in East Asian patients: a retrospective observational study

**DOI:** 10.1186/s12903-023-02933-z

**Published:** 2023-04-28

**Authors:** Adrian Ujin Yap, Ji Woon Park, Jie Lei, Chengge Liu, Seong Hae Kim, Byeong-min Lee, Kai Yuan Fu

**Affiliations:** 1 Center for TMD & Orofacial Pain, Peking University School & Hospital of Stomatology, Beijing, China; 2 Department of Dentistry, Ng Teng Fong General Hospital and Faculty of Dentistry, National University Health System, Singapore, Singapore; 3 National Dental Research Institute Singapore, National Dental Centre Singapore and Duke-NUS Medical School, Singapore Health Services, Singapore, Singapore; 4 Department of Oral Medicine, Seoul National University Dental Hospital, Seoul, Korea; 5 Department of Oral Medicine & Oral Diagnosis, Seoul National University School of Dentistry, 101 Daehak-ro, Jongno-gu, Seoul, Korea; 6 Dental Research Institute, Seoul National University, Seoul, Korea; 7 National Center for Stomatology and National Clinical Research Center for Oral Diseases, Beijing, China; 8 National Engineering Research Center of Oral Biomaterials and Digital Medical Devices, Beijing, China; 9 Department of Dental Biomaterials Science, Seoul National University School of Dentistry, Seoul, Korea

**Keywords:** Temporomandibular disorders, Coronavirus, Pandemic, Pain, Sex, Age

## Abstract

**Background:**

Despite its major existential, societal, and health impacts, research concerning the COVID-19 pandemic and Temporomandibular disorders (TMDs) is still limited. This study examined the effect of the pandemic on TMD subtypes and elucidated the influence of the pandemic, sex, and age on the prospect of pain-related (PT) and/or intra-articular (IT) TMDs in East Asian patients.

**Methods:**

Data were accrued from consecutive new patients attending two university-based TMD/orofacial pain clinics in China and South Korea, 12 months before (BC; Mar 2019-Feb 2020) and during (DC; Mar 2020-Feb 2021) the COVID-19 pandemic. TMD diagnoses were derived from pertinent symptoms, signs, and radiographic findings according to the Diagnostic Criteria for TMDs (DC/TMD) methodology. Patients were subsequently categorized into those with PT, IT, and combined TMDs (CT) and also stratified by attendance period, sex, and age groups (adolescents/young adults [AY] and middle-aged/older adults [MO]) for statistical analyses using Chi-square/Mann-Whitney U tests and logistic regression analyses (α = 0.05).

**Results:**

The BC and DC groups comprised 367 (75.2% females; 82.8% AY) and 471 (74.3% females; 78.3% AY) patients correspondingly. No significant differences in sex and age group distributions were observed. The DC group had significantly more PT/IT conditions with higher prevalence of myalgia, headache, and degenerative joint disease than the BC group. Univariate analyses showed that PT/CT was associated with sex and age, whereas IT was related to the pandemic and age. However, multivariate analyses indicated that the odds of PT were affected by sex (OR = 2.52) and age (OR = 1.04) while the odds of IT (OR = 0.95) and CT (OR = 1.02) were influenced by age only.

**Conclusions:**

The COVID-19 pandemic, as an impact event, did not influence the prospect of PT and/or IT. Sex and age appeared to play more crucial roles in the development of PT and IT/CT respectively.

## Background

Temporomandibular disorders (TMDs) are a cluster of clinical conditions involving pain and/or dysfunction of the stomatognathic system [1, 2]. They are the second most common musculoskeletal problem after chronic low-back pain and TMD symptoms include headaches, temporomandibular joint (TMJ)/masticatory muscle pain, TMJ noises, and jaw movement limitations [1–4]. TMDs affect up to 7% of adolescents and 16% of adults, predominantly females, in the general population [2, 4, 5]. The Diagnostic Criteria for TMDs (DC/TMD) standard classifies common TMDs into pain-related (PT) and intra-articular (IT) conditions [3]. While the main subtypes of PT are arthralgia, myalgia, and headache attributed to TMDs, the main IT subtypes are TMJ disc displacements, degenerative joint disease, and subluxation. TMD pain is the usual reason for treatment-seeking and has been related to poor sleep quality and diminished quality of life (QoL) [2, 6, 7]. The “biopsychosocial” etiology of TMDs is well established and contributing factors encompass gene-environmental interactions, gonadal hormones, poor general health, jaw injuries, oral parafunction, somatization, depression, and anxiety [4, 8, 9]. Patients who seek TMD care, especially those with TMD pain, have been found to have higher prevalence and severity of depression and anxiety [9].

The Coronavirus Disease 2019 (COVID-19), caused by the Severe Acute Respiratory Syndrome Coronavirus 2 (SARS-Cov-2), was first reported in China in December 2019. Due to its rapid spread worldwide, the World Health Organization declared the COVID-19 outbreak a global pandemic in March 2020 [10]. Because of uncertainties about transmission modes, overwhelmed health systems, and the lack of a COVID-19 vaccine during the first year of the pandemic, the measures assumed by most countries involved strict citywide partial-to-total lockdown, social distancing, and “test-trace-isolate-quarantine” (TTIQ) interventions [11]. Though highly effective in breaking transmission chains and reducing COVID-19 deaths, the TTIQ strategy disrupted daily life and had significant economic, existential, societal, and health impacts including elevated levels of depression, anxiety, and stress [12–14]. Given the relationship between TMDs and psychological distress, the latter can induce or aggravate TMD symptoms leading to treatment-seeking [15, 16].

Research concerning TMDs and the COVID-19 pandemic as an “impact event” (phenomena with major societal consequences) is still limited [16–23]. The few cross-sectional studies suggested that the pandemic was associated with greater distress, worse sleep, lower QoL, as well as increased prevalence and intensity of TMD symptoms [16–19]. Psychological distress and prevalence of TMD pain/TMJ sounds escalated further a year after the pandemic [20]. Disparate results were presented by two prospective cohort studies. While one endorsed the negative psychological impact of the pandemic, the other reported no worsening of pain intensity and oral health-related QoL (OHRQoL) in women with painful TMDs [21, 22]. The only case-control study indicated oral parafunction, but not TMD pain increased substantially during the pandemic and women were more distressed than men [23].

As can be seen, further research in this area is warranted. Of interest is the phenotype of TMD patients, particularly TMD subtypes and sex due to their bearing on psychological distress and other variables [4, 5, 8, 9, 24]. Accordingly, the objectives of this study were: (i) to examine the effect of the COVID-19 pandemic on the prevalence of standardized TMD subtypes, (ii) to compare sex and age distributions of East Asian TMD patients seeking care before and during the pandemic, and (iii) to clarify the influence of the pandemic, sex, and age on the prospects of pain-related and/or intra-articular TMDs. The research hypotheses were: (a) the occurrence of painful TMDs increased significantly during the pandemic, (b) sex and age distributions of TMD patients varied substantially between the two attendance periods, and (c) the odds of pain-related TMDs were partial to sex and age, independent of the pandemic.

## Methods and materials

### Study design

This retrospective research was part of a large-scale collaborative study concerning the phenotyping of East Asian TMD patients. This work was approved by the Biomedical Institutional Review Boards at the School of Stomatology, Peking University (PKUSSIRB-201,732,009) and Seoul National University Dental Hospital (ERI22001) in China and South Korea respectively. Informed consent was obtained from all participants or their legal guardians (if under 18 years old) as applicable in China, whilst waiver of consent was granted in South Korea by the Institutional Review Board of Seoul National University Dental Hospital considering the retrospective nature of the study. Data were accrued from consecutive new patients seeking care at two university-based TMD/orofacial pain clinics in Beijing (China) and Seoul (South Korea), 12 months before (March 2019-February 2020) and during (March 2020-February 2021) the COVID-19 pandemic. Using an online sample size calculator (https://www.calculator.net/), a minimum of 197 participants were required for each attendance period based on a 95% confidence level, 5% precision, 48% prevalence of painful TMDs, and pooled yearly estimate of 400 new patients [25]. Study inclusion criteria were complaints of TMD symptoms, age 15–84 years old, and Chinese/Korean language proficiency. Patients with prior orofacial trauma, craniofacial abnormalities, debilitating physical/psychological conditions, drug/substance abuse, cognitive impairments, illiteracy, and incomplete data were excluded. Anamnestic and clinical data were collected at the initial visit as part of routine patient care. Demographic and TMD symptom history were gathered with the Chinese/Korean versions of the DC/TMD Symptom Questionnaire (SQ) (https://ubwp.buffalo.edu/rdc-tmdinternational/) [3].

### TMD diagnosis and subtypes/categories

Clinical examinations were carried out following the DC/TMD protocol by TMD/oral medicine specialists who were formally trained and calibrated in the DC/TMD methodology [3]. Palpation/movement pain, pain location/referral, TMJ noises, as well as jaw movements were assessed and intra-articular disorders were verified with orthopantomography, cone-beam computed tomography (CBCT), and magnetic resonance imaging (MRI), where indicated. Axis I physical diagnoses were derived from pertinent TMD symptoms, signs, and radiographic findings using the DC/TMD algorithms and diagnostic trees. Patients were subsequently categorized into those with pain-related (PT), intra-articular (IT), and combined TMDs (CT; PT plus IT conditions), depending on the presence of the various TMD subtypes. To evaluate the impact of the pandemic, sex, and age on TMD subtypes/categories, the study sample was dichotomized into the following groups: (a) before (BC) and during (DC) the COVID-19 pandemic, (b) females (F) and males (M); (c) adolescents/young adults (AY; aged 15–44 years old) and middle-aged/older adults (MO; aged 45–84 years old) [26].

### Statistical analyses

Data were analyzed utilizing the SPSS statistics software version 27.0 (IBM Corporation, Armonk, New York, USA) with the level of statistical significance set at 0.05. Categorical variables were described as frequencies with percentages and appraised using Chi-square tests. Numerical variables were described as means/medians with standard deviations/interquartile ranges and examined for normality using Shapiro-Wilk’s test. As non-normal distribution was observed, they were appraised using Mann-Whitney U tests. Univariate and multivariate logistic regression analyses were conducted to establish the impact of the pandemic, sex, and age on the prospects of PT, IT, and CT, as well as potential interaction effects between sex and age. Findings were reported as odds ratios (ORs) with 95% confidence intervals (95% CIs).

## Results

Table 1 shows the demographic characteristics of the TMD patients who sought care before and during the pandemic. The BC group comprised 367 patients (mean age 30.86 ± 14.77 years) of which 75.2% were females and 82.8% were adolescents/young adults. Correspondingly, the DC group comprised 471 patients (mean age 33.07 ± 16.02 years) of which 74.3% were females and 78.3% were adolescents/young adults. No significant differences in sex and age group distributions as well as TMD duration were observed between the two groups.

**Table 1:** Demographic characteristics of TMD patients who presented before and during the COVID-19 pandemic

Variables	All patients	Before COVID	During COVID	*P-value*
**Number of TMD patients**				
*n (%)*	838 (100)	367 (100)	471 (100)	
**Sex**				
*Female, n (%)*	626 (74.7)	276 (75.2)	350 (74.3)	0.768
*Male, n (%)*	212 (25.3)	91 (24.8)	121 (25.7)	
*Female;male ratio*	3.0	3.0	2.9	
**Age**				
*Mean (SD)*	32.10 (15.52)	30.86 (14.77)	33.07 (16.02)	0.082^
*Median (IQR)*	27.00 (18.00)	27.00 (15.00)	28.00 (19.00)	
**Age groups**, *n (%)*				
Adolescents/young adults	673 (80.3)	304 (82.8)	369 (78.3)	0.105
Middle-aged/older adults	165 (19.7)	63 (17.2)	102 (21.7)	
**TMD duration (months)**				
*Mean (SD)*	16.80 (37.12)	17.48 (39.89)	16.27 (34.85)	0.520^
*Median (IQR)*	3.00 (11.50)	3.00 (11.50)	3.00 (11.33)	

TMD = temporomandibular disorders; COVID = corona virus disease 2019; SD = standard deviation; IQR = interquartile range

Results of ^Mann-Whitney U and *Chi-square tests

The frequency of TMD subtypes/categories for the BC and DC groups is shown in Table 2. The DC group had significantly higher prevalences of myalgia, specifically local myalgia, and headache as well as more PT conditions than the BC group. In addition, significant differences in the prevalence of degenerative joint disease, number of IT conditions, and total number of TMD conditions were discerned (DC > BC). Concerning TMD categories, the DC group had a significantly lower occurrence of IT than the BC group.

**Table 2:** Frequency of TMD subtypes/categories before and during the COVID-19 pandemic

Variables	All patients	Before COVID (BC)	During COVID (DC)	*P-value*
**Total**				
*n* (%)	838 (100)	367 (100)	471 (100)	
**Pain-related TMDs (PT)**				
*Arthralgia*	374 (44.6)	161 (43.9)	213 (45.2)	0.696*
*Myalgia*	197 (23.5)	60 (16.3)	137 (29.1)	< 0.001* DC > BC
*Local myalgia*	118 (14.1)	32 (8.7)	86 (18.3)	< 0.001* DC > BC
*Myofascial pain*	67 (8.0)	27 (7.4)	40 (8.5)	0.548*
*Myofascial pain with referral*	6 (0.7)	1 (0.3)	5 (1.1)	0.239*
*Headache attributed to TMDs*	51 (6.1)	14 (3.8)	37 (7.9)	0.015* DC > BC
**Number of PT conditions**				
*Mean (SD)*	0.74 (0.78)	0.64 (0.71)	0.82 (0.83)	**0.002^** DC > BC
*Median (IQR)*	1.00 (1.00)	1.00 (1.00)	1.00 (1.00)	
**Intra-articular TMDs (IT)**				
*Disc displacements (DD)*	609 (72.7)	272 (74.1)	337 (71.6)	0.409*
*Disc displacement with reduction (DDwR)*	311 (37.1)	132 (36.0)	179 (38.0)	0.545*
*DDwR*	253 (30.2)	107 (29.2)	146 (31.0)	0.564*
*DDwR with intermittent locking*	58 (6.9)	25 (6.8)	33 (7.0)	0.912*
*Disc displacement without reduction (DDw/oR)*	298 (35.6)	140 (38.2)	158 (33.6)	0.167*
*DDw/oR with limited opening*	128 (15.3)	56 (15.3)	72 (15.3)	0.991*
*DDw/oR without limited opening*	170 (20.3)	84 (22.9)	86 (18.3)	0.098*
*Degenerative joint disease*	259 (30.9)	89 (24.3)	170 (36.1)	< 0.001* DC > BC
Subluxation	14 (1.7)	5 (1.4)	9 (1.9)	0.539*
**Number of IT conditions**				
*Mean (SD)*	1.05 (0.56)	1.00 (0.49)	1.08 (0.61)	**0.022^** DC > BC
*Median (IQR)*	1.00 (0)	1.00 (0)	1.00 (0)	
**Total number of TMD conditions**				
*Mean (SD)*	1.79 (0.91)	1.64 (0.80)	1.91 (0.97)	***< 0.001^*** DC > BC
*Median (IQR)*	2.00 (1.00)	1.00 (1.00)	2.00 (2.00)	
**TMD categories**				
*Pain-related, n (%)*	113 (13.5)	45 (12.3)	68 (14.4)	0.360*
*Intra-articular, n (%)*	363 (43.3)	173 (47.1)	190 (40.3)	0.049* BC > DC
*Combined n (%)*	362 (43.2)	149 (40.6)	213 (45.2)	0.180*

COVID = corona virus disease 2019; SD = standard deviation; IQR = interquartile range

Results of ^Mann-Whitney U and *Chi-square/Z tests. Bold indicates p < 0.05

Tables 3 and 4 indicate the number of TMD conditions and frequency of TMD categories before and during the pandemic for the two sex and age groups.

**Table 3:** Number of TMD conditions and frequency of TMD categories before and during the COVID-19 pandemic according to sex

	Females (F)			Males (M)			Between sexes	
Variables	Before COVID (BC)	During COVID (DC)	*P-value*	Before COVID (BC)	During COVID (DC)	*P-value*	Before COVID *P-value*	During COVID *P-value*
**Number of pain-related conditions**								
*Mean (SD)*	0.64 (0.69)	0.82 (0.85)	**0.024^** DC > BC	0.63 (0.78)	0.82 (0.76)	**0.030^** DC > BC	0.478^	0.775^
*Median (IQR)*	1.00 (1.00)	1.00 (1.00)		0 (1.00)	1.00 (1.00)			
**Number of intra-articular conditions**								
*Mean (SD)*	1.02 (0.47)	1.14 (0.58)	**0.003^** DC > BC	0.93 (0.55)	0.92 (0.67)	0.729^	0.158^	***< 0.001^*** F > M
*Median (IQR)*	1.00 (0)	1.00 (0.25)		1.00 (0)	1.00 (1.00)			
**Total number of TMD conditions**								
*Mean (SD)*	1.66 (0.79)	1.97 (0.99)	***< 0.001^*** DC > BC	1.56 (0.83)	1.74 (0.88)	0.116^	0.132^	**0.028^** F > M
*Median (IQR)*	1.50 (1.00)	2.00 (1.00)		1.00 (1.00)	1.00 (1.00)			
**TMD categories**								
*Pain-related, n (%)*	28 (10.1)	37 (10.6)	0.862*	17 (18.7)	31 (25.6)	0.232*	0.017* M > F	< 0.001* M > F
*Intra-articular, n (%)*	126 (45.7)	146 (41.7)	0.324*	47 (51.6)	44 (36.4)	0.026* BC > DC	0.017* M > F	< 0.001* F > M
*Combined, n (%)*	122 (44.2)	167 (47.7)	0.382*	27 (29.7)	46 (38.0)	0.206*	0.017* F > M	< 0.001* F > M

COVID = corona virus disease 2019; SD = standard deviation; IQR = interquartile range. Results of ^Mann-Whitney U and Chi-square tests. Bold indicates p < 0.05 for before and during COVID comparisons

**Table 4:** Number of TMD conditions and frequency of TMD categories before and during the COVID-19 pandemic according to age groups

	Adolescents/young adults (AY)			Middle-aged/older adults (MO)				Between age groups	
Variables	Before COVID (BC)	During COVID (DC)	*P-value*	Before COVID (BC)	During COVID (DC)		*P-value*	Before COVID *P-value*	During COVID *P-value*
**Number of pain-related conditions**									
*Mean (SD)*	0.57 (0.70)	0.68 (0.79)	0.112^	0.98 (0.68)	1.32 (0.75)	**0.004^** DC > BC		***< 0.001^*** MO > AY	***< 0.001^*** MO > AY
*Median (IQR)*	0 (1.00)	1.00 (1.00)		1.00 (0)	1.00 (1.00)				
**Number of intra-articular conditions**									
*Mean (SD)*	1.02 (0.47)	1.11 (0.57)	**0.026^** DC > BC	0.89 (0.60)	1.00 (0.73)	0.332^		0.055^	0.158^
*Median (IQR)*	1.00 (0)	1.00 (0)		1.00 (0)	1.00 (2.00)				
**Total number of TMD conditions**									
*Mean (SD)*	1.59 (0.76)	1.79 (0.89)	**0.005^** DC > BC	1.87 (0.92)	2.32 (1.12)	**0.011^** DC > BC		**0.017^** MO > AY	***< 0.001***^ MO > AY
*Median (IQR)*	1.00 (1.00)	2.00 (1.00)		2.00 (1.00)	2.00 (2.00)				
**TMD categories**									
*Pain-related, n (%)*	30 (9.9)	41 (11.1)	0.601*	15 (23.8)	27 (26.5)	0.703*		***< 0.001*** * MO > AY	***< 0.001*** * MO > AY
*Intra-articular, n (%)*	160 (52.6)	183 (49.6)	0.433*	13 (20.6)	7 (6.9)	0.008* BC > DC		***< 0.001*** * AY > MO	***< 0.001*** * AY > MO
*Combined, n (%)*	114 (37.5)	145 (39.3)	0.634*	35 (55.6)	68 (66.7)	0.152*		***< 0.001*** * MO > AY	***< 0.001*** * MO > AY

COVID = corona virus disease 2019; SD = standard deviation; IQR = interquartile range. Results of ^Mann-Whitney U and Chi-square tests

Bold indicates p < 0.05 for before and during COVID comparisons

Female and male patients who sought care during the pandemic had considerably more PT conditions than their counterparts who presented before the pandemic. However, only females who sought care during the pandemic had significantly more IT conditions and a greater total number of TMD conditions. No significant differences in TMD categories were detected except for the lower occurrence of IT in male patients presenting during the pandemic. When the sexes were compared, females had significantly more IT conditions and a greater total number of TMD conditions during the pandemic than males. Moreover, they also had notably higher occurrences of IT and CT during this period. Male patients were observed to have a higher occurrence of PT both before and during the pandemic (Table 3).

AY and MO patients who sought care during the pandemic had a significantly greater total number of TMD conditions than their counterparts who presented before the pandemic. While AY patients had significantly more IT conditions, MO patients presenting during the pandemic had considerably more PT conditions. Again, no significant differences in TMD categories were observed apart from the lower occurrence of IT in MO patients presenting during the pandemic. When the age groups were compared, MO patients had considerably more PT conditions, a greater total number of TMD conditions as well as higher occurrences of PT and CT than AY patients at both attendance periods. Conversely, AY patients had a significantly higher prevalence of IT before and during the pandemic (Table 4).

Table 5 displays the results of univariate and multivariate logistic regression analyses for PT, IT, and CT. In the univariate model, PT and CT were associated with sex and age, while IT was related to the pandemic and age. Multivariate analyses indicated that the COVID-19 pandemic did not influence the odds of PT, IT, and CT. The odds of PT were affected by sex (OR = 2.52; 95% CI = 1.65–3.86) and age (OR = 1.04; 95% CI = 1.02–1.05) while the odds of IT were modified by age only (OR = 0.95; 95% CI = 0.93–0.96). The odds of CT were also affected by age (OR = 1.02; 95% CI = 1.01–1.03) with an interaction effect between the female sex and age (OR = 1.02; 95% CI = 1.01–1.02).

**Table 5:** Logistic regression analyses for TMD subtypes

	Univariate		Multivariate	
Variables	Odds ratio (95% CI)	P-value*	Odds ratio (95% CI)	P-value^
**Painful TMDs**				
**Pandemic**				
Before COVID	Reference	*-*	Reference	-
*During COVID*	1.21 (0.81–1.81)	0.361	-	
**Sex**				
*Female*	2.53 (1.67–3.81)	**< 0.001**	2.52 (1.65–3.86)	**< 0.001**
*Male*	Reference		Reference	
**Age**	1.04 (1.02–1.05)	**< 0.001**	1.04 (1.02–1.05)	**< 0.001**
Sex*age				
Female*age			-	**-**
Male*age			Reference	-
**Intra-articular TMDs**				
**Pandemic**				
Before COVID	Reference	-	Reference	-
*During COVID*	0.76 (0.58-1.00)	**0.049**	-	-
**Sex**				
*Female*	0.98 (0.72–1.34)	0.894	-	-
*Male*	Reference		Reference-	-
**Age**	0.95 (0.93–0.96)	**< 0.001**	0.95 (0.93–0.96)	**< 0.001**
Sex*age				
Female*age			-	-
Male*age			Reference-	-
**Combined TMDs**				
**Pandemic**				
Before COVID	Reference		Reference	-
*During COVID*	1.21 (0.92–1.59)	0.180	-	-
**Sex**				
*Female*	1.63 (1.18–2.26)	**0.003**	-	-
*Male*	Reference		Reference	
**Age**	1.03 (1.02–1.04)	**< 0.001**	1.02 (1.01–1.03)	**0.003**
Sex*age				
Female*age			1.02 (1.01–1.02)	**0.001**
Male*age			Reference-	-

COVID = corona virus disease 2019; TMD = temporomandibular disorders; CI = confidence inerval

Results of univariate and multivariate logistic regression analysis. Bold indicates p < 0.05

## Discussion

This retrospective observational study is one of the earliest to examine the effect of “impact events”, specifically the COVID-19 pandemic, on the variance of TMD subtypes in patients with TMDs and elucidate the influence of the pandemic, sex, and age on the prospects of PT and/or IT. The first and third research hypotheses were supported as the prevalence of myalgia/headache was significantly higher during the pandemic and the odds of PT were affected by sex and age. As the differences in sex and age distributions between the two attendance periods were statistically insignificant, the second research hypothesis was not upheld. The systematic translation and use of the evidence-based DC/TMD in this research allowed for standardized history-taking, clinical examination, and rendering of physical TMD diagnoses, enabling the collation of data across the two East Asian sites. TMD attendance increased by about 28% during the COVID-19 pandemic despite the implementation of citywide lockdowns and TTIQ measures. TMD symptoms were usually chronic (> 3 months) and no significant differences in TMD duration were noted between patients who presented before and during the pandemic [27]. The latter suggests that the aggravation of pre-existing TMDs could be the motivator for TMD treatment-seeking.

### TMD subtypes and categories

The frequencies of arthralgia (range 13.0–58.0%), myalgia (range 1.9–50.6%), disc displacements with (range 20.0-44.2%) and without (range 0-12.8%) reduction, as well as degenerative joint disease (range 0-55.6%) were found to vary considerably among TMD patient populations [5]. Apart from disc displacement without reduction (range 33.6–38.2%), the prevalence of TMD subtypes/discrete conditions in East Asian patients were within the documented ranges, both before and during the pandemic. In addition to racial variations, the higher frequency of disc displacements without reduction in this study could be contributed partly by the use of supplementary MRIs for verifying this diagnosis. When compared to MRI, clinical protocols have poor-to-moderate validity for diagnosing disc displacements with and without reduction [28]. Patients who sought care during the pandemic had significantly more PT and IT conditions with higher prevalences of myalgia, headache, and degenerative joint disease than those who presented before the pandemic. As most patients experienced chronic TMDs, the higher frequencies of the aforementioned conditions might be the consequence of intensified TMD pain and/or dysfunction or heightened concerns over TMD problems during the pandemic. Findings were consistent with the elevated levels of psychological distress, oral parafunction, and TMD symptoms reported during the COVID-19 pandemic [18, 19, 23]. Psychological distress was posited to trigger a cascade of events culminating in increased sympathetic activity and release of adreno-corticoid hormones leading to muscle vasoconstriction and amplified stress responses [15]. This coupled with increased awake and/or sleep bruxism during the pandemic could result in sustained masticatory muscle contractions and exacerbation of TMD-related myalgia and headache [15, 18, 19].

### Comparison of sex and age group

Women constituted about three-quarters of all TMD patients in this study. Findings were congruent with the greater risk of women developing TMDs and female-to-male ratios (3.3) reported for other TMD populations [2, 24]. When patients were pooled, 7.76% (65/838), 32.46% (272/838), and 34.49% (289/838) with PT, IT, and CT were females. In contrast, 5.72% (48/838), 10.86% (91/838), and 8.71% (73/838) of all patients with PT, IT, and CT were males. The female predominance has been explained by gender differences in biology (especially sex hormones), social roles, psychological distress, somatization, pain experience, and treatment-seeking behaviors [24]. Though no significant difference was discerned before the pandemic, female patients had significantly more TMD conditions (particularly IT conditions) than male patients during the pandemic. This can be attributed in part to women being more distressed by the pandemic than men, and the accompanying increase in oral parafunctional activities [23]. The larger number of IT conditions also explains the significantly greater occurrence of IT and CT conditions in women. The proportion of male patients with PT was greater than female patients suggesting that TMD pain was the primary reason for treatment-seeking in East Asian men.

Adolescents/young adults formed the majority of TMD patients that had a mean age of 32.1 years. Findings corroborated the age distribution in other East Asian studies and the mean age range (30.2–39.4 years) of TMD patient populations [5, 26, 29]. Both before and during the pandemic, MO patients had substantially more TMD conditions (particularly PT conditions), which also clarifies their significantly greater prevalence of PT and CT, than AY patients. Contrariwise, AY patients had a significantly greater prevalence of IT during the two attendance periods. Findings specify that older and younger patients may be predisposed to TMD pain and dysfunction correspondingly. The underlying mechanism for this phenomenon is unknown and may be the product of experiences and physical changes related to age, especially in females [30, 31]. Population-based studies have also shown a twofold greater prevalence of pain in older adults when compared to younger ones [32]. Even though sex and age appeared to have independent effects on the number of TMD conditions and frequency of TMD subtypes both before and during the pandemic, the two variables could be interlinked and confounding effects may exist. Multivariate analyses were thus performed to adjust for confounders and to explore possible interaction effects between sex and age.

### Influence of the pandemic, sex, and age

While PT and CT were found to be related to sex and age, IT was associated with the pandemic and age in the univariate analysis. After controlling for confounding effects in the multivariate modeling, the odds of PT increased 2.5 folds by being female. Being older increased the odds of PT by 4% and CT by 2%, but reduced the odds of IT by 5%. An interaction effect between sex and age was observed, in which being older and female increased the odds of CT by 2%. Collectively, the results showed that the COVID-19 pandemic, as an impact event, did not consistently influence the prospect of TMD pain and/or dysfunction. Instead, sex and age appeared to play more crucial roles in the development of PT and IT/CT respectively.

Women are known to have more numerous, frequent, and intense somatic symptoms including bodily pains than men. The difference is present in both patient and community-based samples, even with the exclusion of gynecologic or medically unexplained symptoms [24, 33]. Furthermore, they have been reported to have lower pain thresholds/tolerance and a greater ability to discriminate pain which explains partly the markedly greater odds of painful TMDs among female patients [34]. Older females also had marginally greater odds of CT and this was compatible with hormonal changes in women during menopause that is associated with TMJ degeneration and osteoarthritis [31]. Adolescents/young adults had slightly greater odds of IT which was constant with the high prevalence of intra-articular disorders among young people [35, 36]. Purported risk factors for this age group included the female gender, increasing age, awake bruxism, and lip/cheek biting [36].

### Study limitations

The present observational study had several limitations. First, the research only involved East Asian TMD patients and findings cannot be extrapolated to other racial/ethnic groups. The study needs to be repeated in Western and other TMD patient populations before absolute inferences can be made. Second, TMD patients who sought care before and during the pandemic may not be completely identical, ensuing in possible sampling bias. Although this was mitigated by comparable sex and age distributions of the BC and DC groups, variances in psychological distress and other confounding variables may be present but were not investigated. Lastly, even as sex and age were found to affect the prospect of PT and IT/CT, the ,“biopsychosocial” processes involved remain undetermined and warrant further explorations.

## Conclusion

The prevalence of pain-related and/or intra-articular TMDs among East Asian patients was not significantly affected by “impact events”, specifically the COVID-19 pandemic but by sex and age. Being female increased the odds of painful TMDs by 2.5 folds underscoring the importance of considering gender differences in biology and psychosocial characteristics during TMD management as well as TMD service and research planning. While the odds of combined TMDs were marginally increased by being older and female, the odds of intra-articular TMD were increased slightly by being younger. The latter alerts clinicians about the need to screen for TMD signs and symptoms in adolescents/young adults so that early inventions including self-management can be introduced. Intra-articular TMDs, especially TMJ degenerative joint disease, if undiagnosed or left unchecked could result in dentofacial disharmony and bite derangements in addition to functional and psychosocial disabilities. The complex interactions between TMDs and sex-specific experiences/physical changes related to age require further study. Besides cross-sectional investigations, the latter should also involve prospective cohort studies.

## Data Availability

The raw data supporting this study are not in the public domain but are available upon reasonable request from the corresponding author (PJW).
